# Frequency and relevance of psychoeducation in psychiatric diagnoses: Results of two surveys five years apart in German-speaking European countries

**DOI:** 10.1186/1471-244X-13-170

**Published:** 2013-06-18

**Authors:** Christine Rummel-Kluge, Michael Kluge, Werner Kissling

**Affiliations:** 1 Klinik und Poliklinik für Psychiatrie und Psychotherapie am Klinikum rechts der Isar der Technischen Universität München, Munich, Germany; 2 Klinik und Poliklinik für Psychiatrie und Psychotherapie der Universität Leipzig, Semmelweisstr. 10, 04103 Leipzig, Germany

**Keywords:** Psychoeducation, Survey, Schizophrenia, Depression, Interventions

## Abstract

**Background:**

Psychoeducation has been shown to reduce relapse rates in several psychiatric disorders. Studies investigating for which psychiatric diagnoses psychoeducation is offered and assessing its perceived relevance compared to other interventions are lacking.

**Methods:**

A two-part questionnaire addressing these questions was sent to the heads of all psychiatric hospitals in Germany, Austria and Switzerland. Results were compared with those from a similar survey 5 years earlier.

**Results:**

289 of 500 (58%) institutions responded. Significantly (p = 0,02) more institutions (93%) offer any type of psychoeducation as compared to 5 years before (86%). Psychoeducation is mainly offered for schizophrenia (86%) and depression (67%) and less frequently for anxiety disorders (18%) and substance abuse (17%). For the following specific diagnoses it is offered by less than 10% of the institutions: Personality disorder, bipolar disorder, posttraumatic stress disorder, dementia, obsessive compulsive disorder, sleeping disorders, eating disorders, schizophrenia plus substance abuse, pain, attention deficit hyperactivity disorder and early psychosis. 25% offer diagnosis-unspecific psychoeducation. ‘Pharmacotherapy’ (99%), ‘basic occupational therapy’ (95%) and ‘psychoeducation for patients’ (93%) were the therapies being most often, ‘light therapy’ (24%) and ‘sleep deprivation’ (16%) the therapies being least often perceived as relevant by the respondents when asked about the value of different interventions offered in their hospitals. Art therapy (61%) and psychoanalytically oriented psychotherapy (59%), two therapies with a smaller evidence base than light therapy or sleep deprivation, were perceived as relevant by more than the half of the respondents.

**Conclusion:**

Psychoeducation for patients is considered relevant and offered frequently in German-speaking countries, however, mostly only for schizophrenia and depression. The ranking of the perceived relevance of different treatment options suggests that the evidence base is not considered crucial for determining their relevance.

## Background

Psychoeducation is known to reduce relapse rates and readmissions in several psychiatric disorders such as schizophrenia, depression and bipolar disorder [1-4]. Thus, significant mental health expenditure and substantial human suffering can be avoided by the participation of patients and their family members in this low-cost intervention.

Studies assessing for which psychiatric diagnoses psyocheducation is offered are lacking.

The first aim was therefore to investigate for which diagnoses psychoeducation is offered in German speaking psychiatric hospitals and whether there had been changes in these offerings over a time period of five years [5]. The second aim was to assess the perceived relevance of psychoeducation in comparison to other treatment options in psychiatry.

## Methods

### Questionnaire design and definition of psychoeducation

The design and development of the two-part questionnaire (self-completion/self-report with 5 and 26 questions respectively) by the German expert group “Psychoeducational interventions for schizophrenic disorders” [6] has been described in detail in an earlier publication about the first survey carried out in 2004 (Survey I) [5]. In part 1 of both surveys, the perceived relevance of 16 treatment options (e.g. pharmacotherapy or psychoeducation) by the respondents was assessed on a 4-point Likert scale. For analysis, the answers were dichotomized (“none”/”low” and “high”/”very high”). Psychoeducation was defined “as systematic, structured, didactic information on the illness and its treatment, which includes integrating emotional aspects in order to enable the participants to cope with the illness” [6].

### Survey method

Survey II was conducted between December 2008 and August 2009. Part 1, consisting of a 2-page postal questionnaire and a cover letter, was sent to the heads of the departments of all psychiatric hospitals and departments in Germany, Austria and Switzerland (N = 500). The mailing list from the ‘German Hospital Association’ (Deutsche Krankenhaus Gesellschaft) used for Survey I in 2004 was revised. Survey II comprised 500 hospitals whilst Survey I comprised 622 hospitals, mainly due to a reduction in the number of existing hospitals following reorganization of the health care delivery system. The addressed physicians were requested to return the 2-page questionnaire by fax. The questionnaire was sent out again to all non-responders after three and seven months together with a reminder [7]. Part 2 of the survey, which consisted of a more detailed questionnaire, was sent directly to those individuals designated as responsible for diagnosis-specific psychoeducational groups by the respondents to Part 1 of the survey.

### Analyses

Responses to the two surveys were analyzed using descriptive statistics. Pearson's chi-squared tests were used to compare changes in the range of psychoeducation offered and in the perceived relevance of the various interventions. All calculations were done with PASW Statistics 18 Version 18.0.0.

As this study was a survey asking for the professional opinion of the participating professionals (mainly physicians and psychologists), and as no definite data on patients was solicited, ethical approval was not required according to the regulations of the countries where the study was conducted (Swiss Working Group of Ethics committees, rules of procedure of the Ethics committee of the Medical University of Innsbruck).

## Results

### Respondents

In Survey II, 289 of the 500 questionnaires constituting Part 1 of the survey (response quota 58%) were returned after the initial request or after one or two reminders. 210 respondents were male (76%). 163 respondents were head of the department (58%), 77 were consultant psychiatrists (27%), 28 were psychologists (10%) and 13 were designated “other” (e.g. psychiatry residents) (5%). 115 respondents worked in the psychiatric departments of general hospitals (40%), 83 in psychiatric state hospitals (29%), 35 in psychiatric university hospitals (12%) and 56 in other institutions (19%).

### Conduction of psychoducation

Ninety-three percent (93%) of the respondents to Survey II (2009) reported that psychoeducation as defined in the cover letter had been conducted in their institution. This was a statistically significant increase compared to 86% in Survey I (2004) (X^2^=5.44, p=0.020) (Table 1). For the specific diagnoses, statistically significant increases were found for depression, bipolar disorder and anxiety disorder. Newly listed diagnoses for which psychoeducation was offered were ‘schizophrenia and substance abuse’, ‘pain’, ‘attention-deficit/hyperactivity disorder’ and ‘early psychoses’.

**Table 1:** Conduction of psychoeducation for different diagnoses in 2004 and 2009

	**2004**	**2009**		
	**(n=337)**	**(n=288)**	**X**^**2**^**-test**^**1**^	***p-value***
	**(%)**	**(%)**		
**Any type of Psychoeducation**	86	93	5.44	**0.02**
**Schizophrenia**	84	86	0.28	0.60
**Depression**	59	67	4.714	**0.042**
**Substance Abuse**	17	17	0.004	0.91
**Anxiety Disorder**	9	18	7.73	**0.005**
**Personality Disorder**	7	8	0.006	0.94
**Bipolar Disorder**	4	9	6.02	**0.014**
**PTSD**	3	0.4	5.79	**0.02**
**Dementia**	3	3	0.006	0.94
**OCD**	2	1	1.13	0.29
**Sleeping Disorders**	1	2	0.92	0.34
**Eating Disorders**	1	1	0.24	0.63
**Schizophrenia and Substance abuse**	0	3	n. a.	n. a.
**Pain**	0	2	n. a.	n. a.
**ADHD**	0	1	n. a.	n. a.
**Early Psychosis**	0	0.6	n. a.	n. a.
**Diagnosis-unspecific Psychoeducation**	23	25	0.45	0.50

^1^degrees of freedom = 1, n.a. = not applicable.

### Reasons for not offering psychoeducation

Seven percent (n=19) of the respondents to Survey II (2009) reported that no psychoeducation whatsoever had been conducted at their institution. ‘Lack of manpower’ (28%), ‘lack of time’ (22%) and ‘lack of know-how’ (17%) were the most frequent reasons given for this situation. Consequently, ‘additional staff’ (39%), but also a ‘participation fee’ (44%) were reported to be necessary before being able to start to offer psychoeducational groups.

### Comparison of relevance of different therapeutic options

‘Pharmacotherapy’, ‘basic occupational therapy’ and ‘psychoeducation for patients’ were the three therapeutic options that were perceived as being the most relevant by the respondents in both surveys (Table 2). ’Psychoeducation for patients’ was significantly (p=0.035) more often considered ‘relevant’ or ‘very relevant’ in Survey II (93%) than in Survey I (87%). Other statistically significant changes were found for behavioural therapy - an increase both for individual therapy and group therapy sessions, an increase for electroconvulsive therapy and a decrease for sleep deprivation. The relevance of ‘psychoeducation for families’ did not change significantly; it was ‘high’ or ‘very high’ for 59% of the respondents in Survey II.

**Table 2:** High or very high perceived relevance of treatment options in 2004 and 2009

	**2004**	**2009**		
	**(n=295)**	**(n=246)**	**X**^**2**^**-test**^**1**^	***p-value***
	**(%)**	**(%)**		
**Pharmacotherapy**	97	99	2.92	0.11
**Occupational Therapy (day structuring oriented)**	93	95	1.38	0.29
**Psychoeducation for patients**	87	93	4.71	**0.035**
**Sociotherapy**	87	85	0.36	0.62
**Behavioural Therapy (single)**	76	85	7.02	**0.01**
**Behavioural Therapy (group)**	73	83	9.08	**0.003**
**Physiotherapy**	72	74	0.33	0.63
**Psychoanalytic oriented psychotherapy (single)**	65	59	2.24	0.16
**Occupational Therapy (work oriented)**	60	54	1.83	0.19
**Art Therapy**	58	61	0.30	0.60
**Psychoeducation for families**	57	59	0.09	0.79
**Psychoanalytic oriented psychotherapy (group)**	57	56	0.08	0.80
**Music Therapy**	53	55	0.13	0.73
**Sleep Deprivation**	24	16	4.71	**0.03**
**Light Therapy**	20	24	1.55	0.26
**Electroconvulsive Therapy**	18	30	10.8	**0.001**

^1^degrees of freedom = 1.

## Discussion

The perceived relevance of psychoeducation for patients is high in German-speaking European countries. Psychoeducation is commonly offered and there has been a statistically significant increase in the use of this intervention from 2004 (Survey I) to 2009 (Survey II). Psychoeducation of any type is now offered in 93% of the psychiatric hospitals that responded to the survey. However, only psychoeducation for schizophrenia (86%) and depression (67%) is common, whereas it is much less common for all other specific diagnoses (e.g. for anxiety disorder 18%) Diagnosis-unspecific psychoeducation is offered on an ongoing basis in about a quarter of the hospitals. This method takes into account the fact that smaller hospitals may have only few patients with a particular diagnosis. As a result, the most frequently declared obstacle for providing psychoeducation is lack of manpower and lack of time [8].

Compared to Survey I in 2004, statistically significantly more psychoeducation is now offered to patients with depression, bipolar disorder and anxiety disorder, for which several recent guidelines or reviews now include psychoeducation as an effective intervention and clinical trials have shown its efficacy [9-12].

It is important to note, however, that whilst the perceived significance for psychoeducation for patients was high (93%), the significance for psychoeducation was perceived as being only modest for families (59%). This is surprising, as there is empirical evidence that psychoeducational programs for family members for several psychiatric conditions are in fact effective [2,13-17]. Furthermore, the perceived relevance of psychoeducation for psychiatric patients in general significantly increased from 2004 (Survey I) to 2009 (Survey II), whereas there was no significant change in terms of the perceived relevance of family psychoeducation.

This result is in line with one of the most intriguing finding of the study: The evidence base of the different therapeutic options or interventions is quite obviously *not* the crucial criterion for determining their relevance in the eyes of the heads of psychiatric institutions in German-speaking countries in Europe. Therapies with rather low evidence base (e. g. psychoanalytically oriented psychotherapy [18] or art therapy [19]) were considered to be ‘relevant’ or ’very relevant’ 2 to 4 times more often compared to several interventions with a better evidence base such as light therapy [20] or sleep deprivation [21]. This is particularly interesting since these therapies are easy to carry out and are usually tolerated well. However, it is difficult to generalize here because the evidence base for specific treatment options varies between the different diagnoses.

There are three main limitations to this study. First, not all addressed physicians responded to the survey. However, the response rate (58%) was similar or greater than those of comparable surveys [22-24]. Secondly, due to changes in the health care delivery systems in the German-speaking countries in Europe, the absolute number of hospitals that were invited to participabte was lower in Survey II than in Survey I. However, response rates were comparable. Thirdly, answering the questions in a socially desired manner might have contributed to the high perceived relevance of psychoeducation compared to other treatment options or interventions, as psychoeducation was the main focus of the two surveys.

## Conclusions

Psychoeducation for patients is considered relevant and offered frequently in German-speaking countries in Europe. However, psychoeducation is common only for the diagnoses schizophrenia and depression. The ranking of the perceived relevance of different therapeutic options or interventions indicates that the evidence base is not considered to be the crucial criterion for determining their relevance.

## Competing interest

The authors declare that they do not have competing interests.

## Authors’ contributions

All authors were involved in the analysis and interpretation of the data in this manuscript and were involved in the drafting and revising of the manuscript for intellectual content. All authors read and approved the final manuscript.

## Pre-publication history

The pre-publication history for this paper can be accessed here:

http://www.biomedcentral.com/1471-244X/13/170/prepub
